# Spin filtering in self-assembled bowl-shaped aromatics†

**DOI:** 10.1039/d5sc01660f

**Published:** 2025-04-21

**Authors:** Jorge Labella, Anu Gupta, Anil Kumar, Elisa López-Serrano, Deb Kumar Bhowmick, Ron Naaman, Tomás Torres

**Affiliations:** a Department of Molecular Engineering, Kyoto University Katsura Nishikyo-ku Kyoto 615-8510 Japan labella.jorge.54u@st.kyoto-u.ac.jp; b Department of Organic Chemistry, Universidad Autónoma de Madrid, Campus de Cantoblanco C/ Francisco Tomás y Valiente 7 28049 Madrid Spain tomas.torres@uam.es; c Department of Chemical and Biological Physics, Weizmann Institute of Science 7610001 Israel ron.naaman@weizmann.ac.il; d Institute for Advanced Research in Chemical Sciences (IAdChem), Universidad Autónoma de Madrid 28049 Madrid Spain; e IMDEA-Nanociencia, Campus de Cantoblanco 28049 Madrid Spain

## Abstract

Bowl-shaped aromatics can be chiral, so their columnar self-assembly can yield homochiral supramolecular structures. This valuable—but underexploited—property adds a novel twist to the disruptive applications of these materials, rendering them potentially useful in the burgeoning field of molecular spintronics. Herein, we demonstrate that columnar arrays based on chiral subphthalocyanine aromatics can efficiently function as spin filters *via* the chirality-induced spin selectivity (CISS) effect. To this end, we prepared enantiopure SubPcs engineered to establish head-to-tail intermolecular interactions with varying strengths, as reflected in their final self-assembled structures. Specifically, SubPcs equipped with peripheral arylamides and long paraffinic chains form fibers, whereas those endowed solely with aromatic moieties featuring paraffinic chains lead to the formation of nanorings. Using magnetic-conductive atomic force microscopy (mc-AFM), we observed that these supramolecular architectures display effective spin filtering, yielding spin polarization values between 35% and 45%. Overall, these findings open a new frontier in the application of columnar materials based on π-conjugated bowl-shaped derivatives.

## Abstract 2

Magnetic-conductive AFM reveals that self-assembled bowl-shaped aromatics function as molecular spin filters.

## Graphical abstract

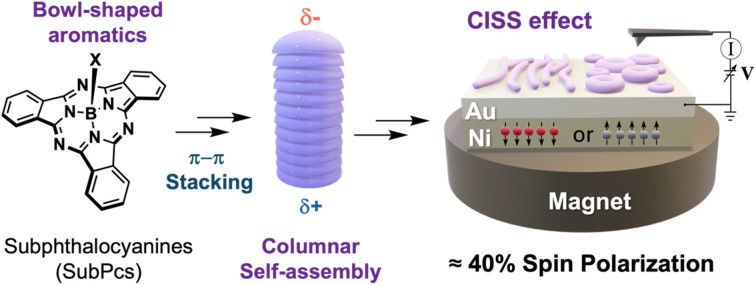

## Introduction

**Fig. 1:**
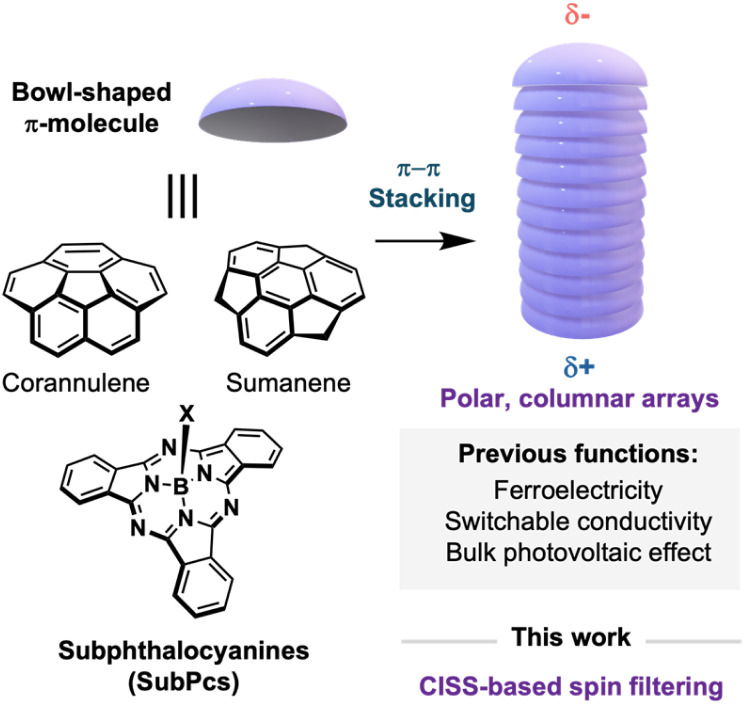
Representative examples of bowl-shaped aromatics and a representation of their columnar self-assembly.

Disrupting the planarity of π-conjugated molecules in the form of bowl-shaped geometries has emerged as an appealing strategy in the design of advanced π-organic materials.^1^ Due to their non-centrosymmetric nature, bowl-shaped aromatics develop a pronounced dipole moment along the molecular axis and exhibit a topology that promotes the formation of columnar arrays through electronically complementary concave–convex interactions (Fig. 1).^2^ Consequently, these molecules serve as unique building blocks for constructing highly ordered, polarized columnar architectures—a rare type of supramolecular assembly with applications in cutting-edge technologies such as ferroelectrics,^3^ nonlinear optics,^4^ polarized semiconductors,^5^ and the bulk photovoltaic effect.^6^

A highly valuable property that remains underexploited in columnar arrays built from bowl-shaped aromatics is chirality, which is at reach just by proper functionalization of the building block periphery.^7^ Chiral π-conjugated organics have attracted widespread attention in spintronics since it has been widely demonstrated that certain chiral organic molecules can generate spin-polarized currents when electrons traverse them.^8^ This remarkable phenomenon, known as chirality-induced spin selectivity (CISS),^9^ holds tremendous potential to reduce noise and energy consumption during information transfer and processing, and it can be also exploited in diverse fields ranging from catalysis^10^ and enantio-separation^11^ to long-range electron transfer^12^ and chirality induction.^13^

The CISS efficiency is measured by the spin polarization (SP) value, which quantifies the relative abundance of electrons with one spin orientation compared to those with the opposite. In order to maximize this value and produce highly spin-polarized currents, it is crucial to organize the enantiopure molecules into well-defined supramolecular assemblies exhibiting long-range chirality.^9^ Such order can be achieved through multiple strategies, with the formation of columnar supramolecular polymers being among the most powerful.^14,15^ For example, the work developed by Meijer's group and some of us demonstrated spin polarizations (SPs) of up to 80% by employing porphyrins equipped with peripheral chiral chains acting as chirality inductors.^14^

Inspired by this precedent, we envisioned that chiral columnar arrays composed of bowl-shaped aromatics could serve also as spin-filtering systems, thereby adding another functionality to the toolbox of these unique materials. Herein, we explore the CISS effect of enantiopure columnar assemblies constructed *via* the self-assembly of bowl-shaped aromatics. Specifically, by exploiting the high configurational stability and polarity of subphthalocyanines (SubPcs), well-known bowl-shaped porphyrinoids, we prepared supramolecular polymers whose structural flexibility is tuned by modulating the intermolecular interactions established between the self-assembly units. These assemblies were integrated into devices by simple spin-coating, and the spin filtering capability was measured using magnetic conductive probe atomic force microscopy (mc-AFM). Notably, these assemblies exhibit SP values of approximately 35–45% at room temperature, which is comparable to efficient spin filters reported in the literature. The results herein reported will expand the applications of bowl-shaped aromatics and pave the way for the development of multifunctional spintronic materials.

## Results and discussion

Among the few examples of chiral bowl-shaped π-molecules reported in the literature,^1^ SubPcs hold a privileged position due to their exceptional structural and photophysical properties.^16–18^ Unlike other well-known bowl-shaped π-systems, such as corannulenes or sumanenes, SubPcs feature a rigid skeleton in which bowl-to-bowl inversion is inhibited, thereby conferring configurational stability,^19^ a property fundamental to the development of stable chiral materials. Their inherent rigidity also makes them ideal scaffolds for constructing robust columnar materials that exhibit unique optoelectronic characteristics, including strong absorption and emission in the visible range, ambipolar behavior, and pronounced polarization.^20–23^ Furthermore, we have recently demonstrated that simple SubPcs can function as effective spin filters^24^ On this basis, we selected SubPcs as ideal candidates for this study.

**Fig. 2:**
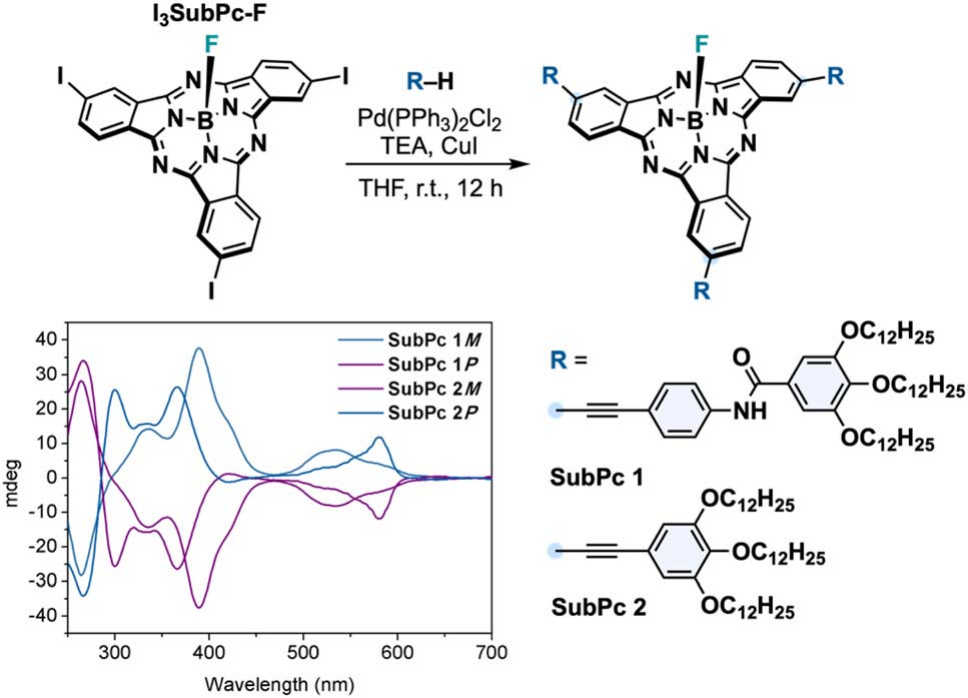
Synthetic route to **SubPc 1–2** and their CD spectra in THF ([SubPc] = 3.2 × 10^−5^ M).

Fig. 2 shows the self-assembly units used to construct the columnar assemblies in this study. The selected SubPcs feature peripheral substituents designed to promote intermolecular interactions that drive concave–convex packing. In addition, fluorine was installed as an axial ligand because it is both small and polar—two indispensable attributes for the head-to-tail linking of SubPcs. SubPc 1 incorporates benzamide fragments to stablish intermolecular interactions *via* H-bonding **SubPc 1**, whereas SubPc 2 just features alkynylbenzene substituents with long paraffinic chains. Both SubPcs have been previously shown to form columnar assemblies in liquid-crystalline^22^ and solution phases.^23^ As depicted in Fig. 2, both compounds were synthesized from a *C*_3_-symmetric iodinated precursor (I_3_SubPc-F) *via* Sonogashira cross-coupling with the corresponding alkynes. I_3_SubPc-F was obtained either as a racemic mixture or as enantiopure (*M* or *P*) following a procedure recently developed by our group.^23^ The enantiopure derivative was obtained *via* HPLC resolution prior to axial exchange. The enantiopurity of *M* and *P* enantiomers of SubPcs 1-2 (SubPc *M*/*P*-1 and SubPc *M*/*P*-2) was confirmed by HPLC and in-solution CD (Fig. 2 and ESI†).

**Fig. 3:**
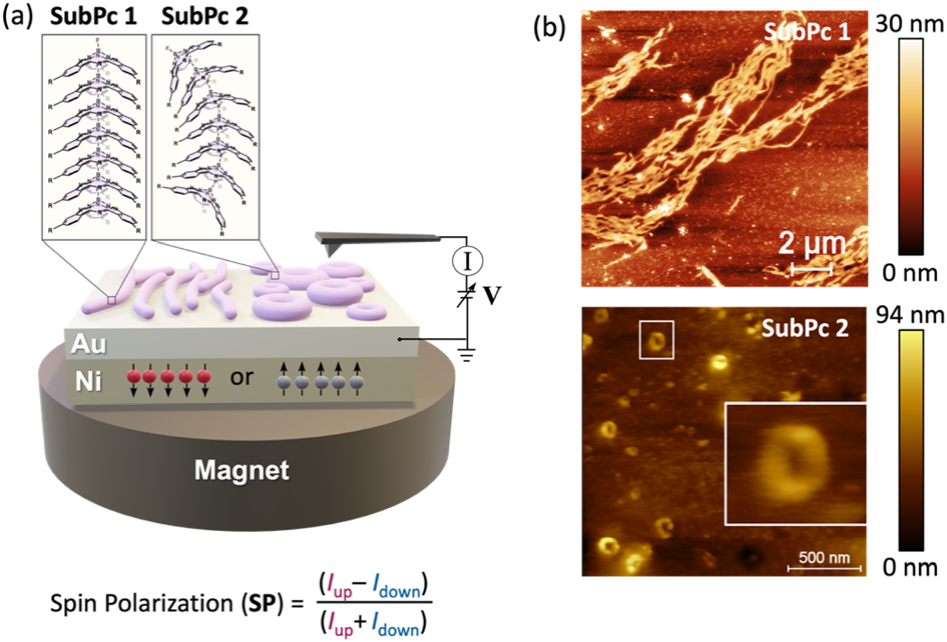
(a) Schematic illustration of the mc-AFM setup used to assess the CISS effect in this work. The pink circular and linear forms represent the experimentally observed fibers and nanorings, respectively. The magnified view illustrates a proposed head-to-tail stacking arrangement within the self-assemblies. (b) AFM images of surfaces spin-coated with self-assembled SubPcs 1 and 2.

A variety of techniques have been developed to assess spin-selective transport through molecules, including photoelectron spectroscopy,^25^ current-perpendicular-to-plane (CPP) magnetoresistance (MR),^26^ spin-polarized conductive atomic force microscopy,^27^ Hall voltage detection,^28^ or current-in-plane magnetoresistance (CIP-MR).^29^ In our study, we primarily employ magnetic-conductive atomic force microscopy (mc-AFM) because of its experimental simplicity, reliability, and compatibility with a wide range of organic compounds.^30^ A schematic representation of the mc-AFM setup is shown in Fig. 3a. In our configuration, the enantiopure molecule is deposited on a gold-coated nickel substrate (Au/Ni) that is magnetized perpendicularly to its surface, with the north pole oriented either upward or downward. The external magnetic field thus determines the spin orientation of electrons emitted from the nickel substrate as they traverse the deposited chiral material. An electric potential is applied so that the AFM tip is maintained at ground while the potential on the Au/Ni substrate is varied. In this way, spin-selective electron transport is readily measured and quantified by the SP value, defined as the relative difference between the currents measured under the two magnetization configurations, *i.e.*, SP = (*I*_up_ − *I*_down_)/(*I*_up_ + *I*_down_)] × 100.

The SubPcs were deposited onto an Au/Ni substrate *via* spin-coating from a solution in methylcyclohexane (MCH), a solvent known to promote self-assembly. The formation of columnar arrays is particularly evident for SubPc 1, as UV-vis absorption spectra reveal a pronounced blue shift in the SubPc Q band—a diagnostic signature of H-type aggregate formation.^23^ In contrast, SubPc 2, which lacks the capacity to establish robust intermolecular interactions, does not exhibit this behavior at comparable low concentrations. AFM analysis (Fig. 3b) revealed marked differences between the two compounds. While SubPc 1 forms arrays of well-defined fibers consistent with the dimensions expected for individual molecular assemblies,^23^SubPc 2 organizes into nanorings (widths in the range of 80–100 nm) whose heights arise from the stacking of multiple SubPc columnar arrays. This organization probably arises from the absence of strong directional hydrogen bonding, which renders the columnar packing of SubPc 2 more susceptible to distortion, ultimately leading to the formation bent structures and, eventually, to closed structures.

**Fig. 4:**
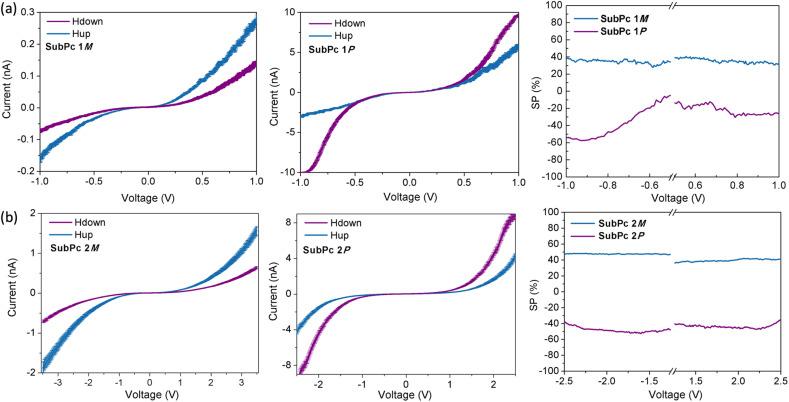
Spin-dependent transport properties measured by mc-AFM for (a) SubPc 1 and (b) SubPc 2. From left to right, the averaged current–voltage (*I*–*V*) curves recorded for the *M* and *P* enantiomers, followed by the spin polarization percentage (SP%) as a function of the applied bias. The blue line represents the current when the magnet's north pole is oriented upward relative to the substrate, and the purple line corresponds to the current when the magnet's north pole is oriented downward.

The results obtained from the mc-AFM experiments on fibers and nanorings composed of SubPc 1 and SubPc 2, respectively, are presented in Fig. 4. As shown in the current–voltage (*I–V*) curves, the measured current for both the *M* and *P* enantiomers of SubPc 1 and SubPc 2 strongly depends on the magnetization direction of the substrate. Specifically, the *M* enantiomers exhibit higher currents when the substrate is magnetized with the magnetic field oriented upward, whereas the *P* enantiomers display lower currents under the same condition. Each curve represents an average of 50 individual measurements. As expected, the racemic compound does not exhibit any spin preference (see ESI†), confirming that both enantiopure SubPcs 1 and 2 display spin-selective charge transport. We subsequently quantified these spin-filtering properties by calculating the SP percentage as a function of the applied voltage (Fig. 4). Remarkably, average SP values of approximately +35 ± 5% for and +40 ± 5% were obtained for SubPc 1 and SubPc 2, respectively. These spin filtering is comparable to those reported for other supramolecular polymers. It should be noted, however, that these SP values are slightly lower than those observed for thin films based on tribrominated SubPcs,^24^ which, although capable of forming well-ordered nanostructures, are less prone to organizing into columnar stacks than SubPc 1 and 2. This suggests that, in the solid state, tribrominated SubPcs form a chiral structure that is more efficient for spin-selective charge transport than the self-assemblies described herein. Importantly, upon columnar organization, the CD signal of the SubPcs slightly decreases compared to that of dissolved SubPc monomers,^23^ which may account for the lower spin-filtering efficiency observed given the direct correlation between the CD signal and spin selection efficiency.^31^ It is worth highlighting that, in this particular case, the spin filtering does not depend significantly on the morphology of the nanostructures formed. This observation leads us to hypothesize that both SubPc 1 and 2 adopt a head-to-tail stacking mode, which—although more or less prone to bending—results in a similar chiral organization.

## Conclusions

We have demonstrated the CISS effect in self-assembled chiral bowl-shaped aromatics, underscoring their significant potential for spintronic applications. Using mc-AFM, we showed that enantiopure SubPcs—self-assembling into either nanorings or columnar structures on metallic surfaces—can function as effective spin filters, achieving spin polarizations of approximately 40%. Although further efforts are needed to enhance these spin polarization values, our study introduces an additional valuable property of columnar arrays based on bowl-shaped aromatics: spin selection. We are confident that this work will stimulate the development of novel chiral assemblies and supramolecular polymers with optimized morphologies, thereby advancing the potential of bowl-shaped aromatics, which remain in an early yet promising technological stage.

## Data availability

The data supporting the findings of this study is available within the article and its ESI.†

## Author contributions

J. L., R. N. and T. T. designed the research. J. L. and E. L.-S. performed the synthesis. A. G., A. K. and D. K. B. performed the mc-AFM measurements. J. L., A. G. and A. K. conducted the analysis of the results. All authors contributed to the writing and editing of the paper. Overall, J. L., A. G. and D. K. B. contributed equally to this publication.

## Conflicts of interest

There are no conflicts to declare.

## Supplementary Material

SC-016-D5SC01660F-s001
