# Supplementary material for: Spin filtering in self-assembled bowl-shaped aromatics
Source: Chem Sci. 2025 Apr 21;16(20):8783–7. doi: 10.1039/d5sc01660f (PMC12013725; doi:10.1039/d5sc01660f)
Supplement: SC-016-D5SC01660F-s001 [file SC-016-D5SC01660F-s001.pdf]

Supplementary Information (SI) for Chemical Science.
This journal is © The Royal Society of Chemistry 2025

Supplementary Information

Spin Filtering in Self-Assembled Bowl-Shaped Aromatics

Jorge Labella,[*‡a] Anu Gupta,[b‡] Anil Kumar,[b‡] Elisa López-Serrano,[a] Deb Kumar Bhowmick,[b] Ron Naaman, [*b] Tomás Torres[* a,c,d].

[a] Departamento de Química Orgánica, Universidad Autónoma de Madrid, Madrid, 28049, Spain.

[b]Department of Chemical and Biological Physics, Weizmann Institute of Science, 7610001, Israel.

[c] Institute for Advanced Research in Chemical Sciences (IAdChem), Universidad Autónoma de Madrid, Madrid, 28049, Spain.

[d] IMDEA-Nanociencia, Campus de Cantoblanco, Madrid, 28049, Spain.

[‡] These authors contributed equally to this work.

1. Synthesis and characterization of Chiral SubPc

Chemicals were purchased from commercial suppliers and used without further purification unless otherwise stated. Thin layer chromatography (TLC) was carried out employing aluminium sheets coated with silica gel type 60 F254 (0.2 mm thick, E. Merck). Purification and separation of the C_1 and C_3 regioisomers of the synthesized SubPcs was performed by column chromatography using silica gel (230–400 mesh, 0.040–0.063 mm, Merck). Matrix Assisted Laser Desorption/ Ionization-Time of Flight (MALDI-TOF) spectra were obtained using a Bruker Ultraflex III spectrometer equipped with a Nd-YAG laser operating at 337 nm. High Resolution Mass Spectrometry (HR-MS) spectra were recorded employing ESI Positive Q-TOF using a Bruker Maxis II. The different matrixes employed are indicated for each spectrum. Mass spectrometry data are expressed in m/z units. ^{1}H-NMR spectra were recorded on a Bruker AC-300 (300 MHz) at room temperature. In the ^{1}H-NMR spectra, the chemical shifts (δ) are measured in ppm relative to the correspondent deuterated solvent. UV-Vis spectra were recorded employing a JASCO-V660 spectrophotometer. CD spectra were recorded with a JASCO V-815 equipment. Resolution of racemic SubPcs was carried out by chiral HPLC using an Agilent 1200 equipment with a semi-preparative Daicel Chiralpak IC column (10 mm ø x 20 mmL).[1] As detailed in the manuscript, **SubPc 1** and **SubPc 2** were prepared via Sonogashira cross-coupling with the corresponding alkynes. Both the racemic and enantiopure versions of **SubPc 1** were obtained following the procedure described in ref. 1, whereas those of **SubPc 2** were prepared according to ref. 2. HPLC analysis confirmed the enantiopurity (see below).

SubPc 1

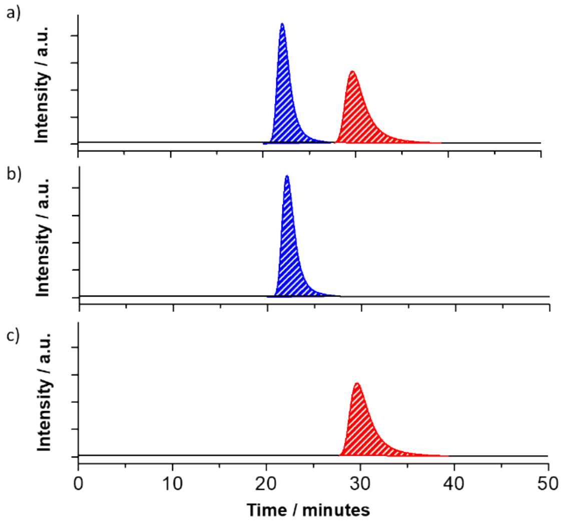

Figure S1.1. a) HPLC chromatogram of a racemic mixture of **SubPc 1** with peaks corresponding to the *M* (blue trace) and *P* (red trace) enantiomers. The percentage area underneath the first and second peak is 50.0% in both cases. b-c) HPLC chromatogram of the corresponding pure enantiomers. Eluting solvents = toluene/hexane/AcOEt 60:30:10; flow rate = 1 mL min^{-1}; temperature = 20 ºC, detection wavelength = 560 nm.

1. J. Labella, E. López-Serrano, D. Aranda, M. J. Mayoral, E. Ortí and T. Torres, *Chem. Sci.,* 2024, **15**, 13760–13767.
2. J. Guilleme, J. Arago, E. Orti, E. Cavero, T. Sierra, J. Ortega, C. L. Folcia, J. Etxebarria, D. Gonzalez-Rodriguez and T. Torres, *J. Mater. Chem. C,* 2015, **3**, 985–989.

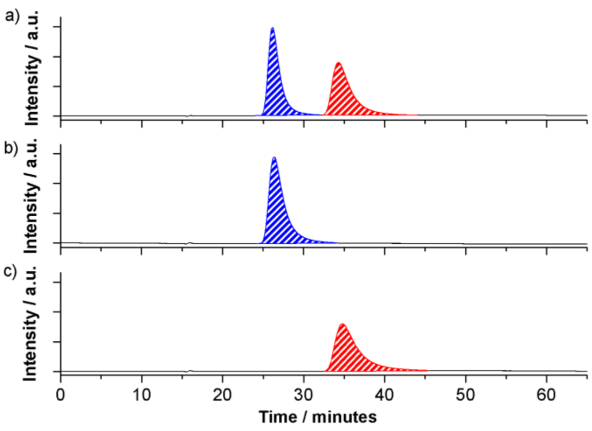

Figure S1.1. a) HPLC chromatogram of a racemic mixture of **SubPc 2** with peaks corresponding to the *M* (blue trace) and *P* (red trace) enantiomers. The percentage area underneath the first and second peak is 50.0% in both cases. b-c) HPLC chromatogram of the corresponding pure enantiomers. Eluting solvents = toluene/hexane 60:40; flow rate = 0.8 mL min^{-1}; temperature = 10 ºC, detection wavelength = 560 nm.

2. Thin-film preparation and magnetic-conductive atomic force microscopy (mc-AFM) experiments.

Thin-film preparation

The substrates for the mc-AFM studies were prepared using the e-beam evaporation deposition technique on Si (100) wafer. An 8 nm Ti layer followed by a 60 nm layer of Ni layer and a 8 nm layer of Au layer was deposited on a Si wafer. Thin gold layers were deposited to protect Ni layer from oxidizing. The deposited multilayer surfaces were cleaned by immersing them first in acetone and then in ethanol for 10 minutes. The role of the Ni/Au surfaces is to allow spin polarization of electrons injected from the surface into the chiral molecules induced by a magnetic field. For **SubPc 1** sample preparation, 1 mg/ml of the corresponding SubPc was prepared in methylene chloride (DCM) and sonicated for 1 minute at room temperature. Then, 17 µL of the solution was transferred to another vial and dried using air flow. Then, 2 mL of methylcyclohexane were added and sonicated for 15 minutes at room temperature. The as prepared solution was spin-coated on the Ni/Au surfaces at 2000 rpm for 40 sec. The **SubPc 2** layer were deposited on the Ni/Au surfaces by spin coating the substrate with a corresponding compound solution of 0.5 mg /ml in toluene at 4000 rpm for 40 sec. Further, substrates were heated at a temperature of 125°C for 10 min and cooling substrates to room temperature at a cooling rate of 10°C/min.

mc-AFM experiments:

Magnetic field-dependent current-voltage (I-V) characteristics of the prepared samples were determined using a multimodal scanning magnetic probe microscopy (SPM) system equipped with a Beetle Ambient AFM and an electromagnet with an R9 electronic controller (RHK Technology). Voltage spectroscopy for the I-V measurements was performed by applying voltage ramps with a non-magnetic Pt tip (DPE-XSC11, µmasch with spring constant 3-5 Nm-1) in contact mode. At least 50 I-V curves were scanned in an applied magnetic field of 0.50 T for both magnetic field orientations (field UP and DOWN), and representative plots for (P) and (M) enantiomers of **SubPcs 1–2** on Ni/Au surfaces are depicted in Figure S1 and S2, respectively.

Spin dependent transport properties for (M) and (P) enantiomers on Ni/Au surfaces:

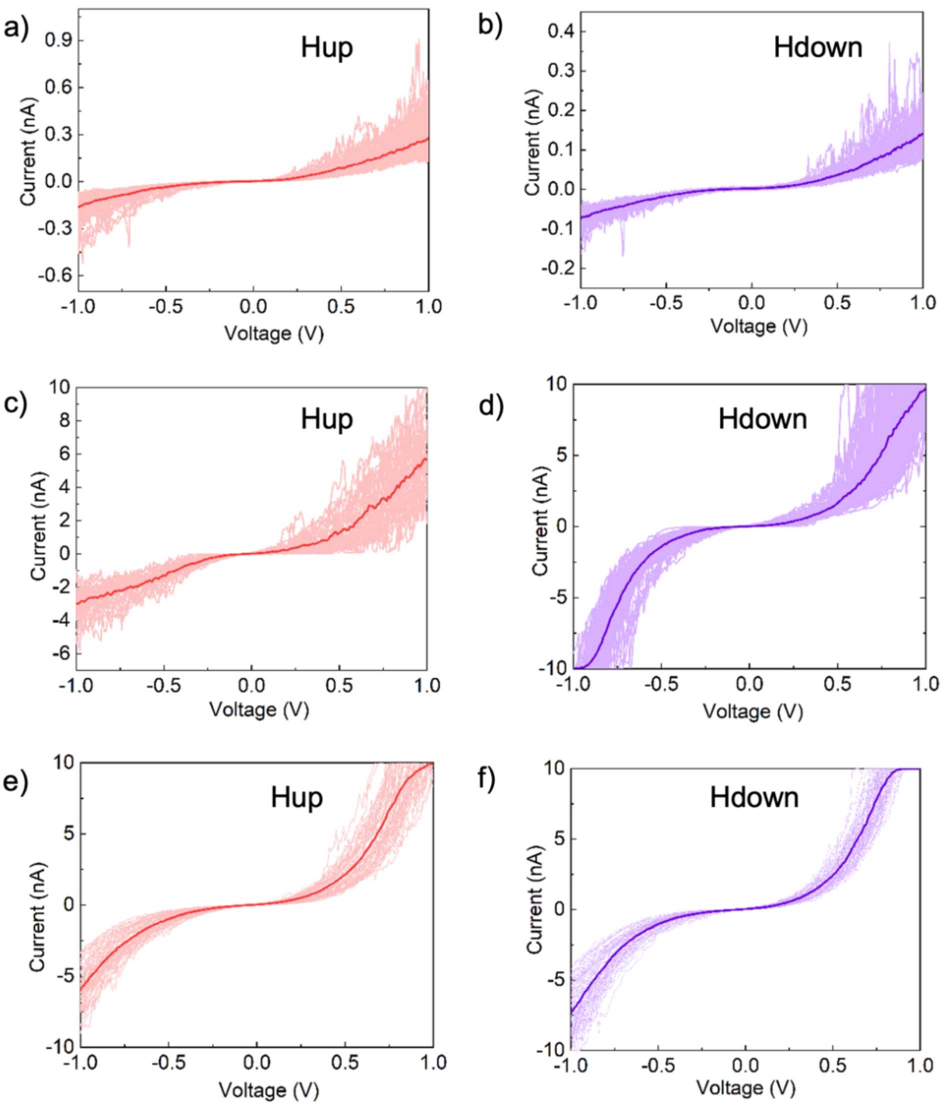

Figure S1. Current versus voltage (I-V) plots recorded for spin-coated of **SubPc 1** on Ni-Au substrates. (a-b) ***M*** enantiomer, (c-d) ***P*** enantiomer, (e-f) racemic mixture. In all cases, the Ni substrates were magnetized with the north pole pointing in the up or down orientations as indicated each case. The width of the lines (in multiple colours) represents the standard deviation of the measurements.

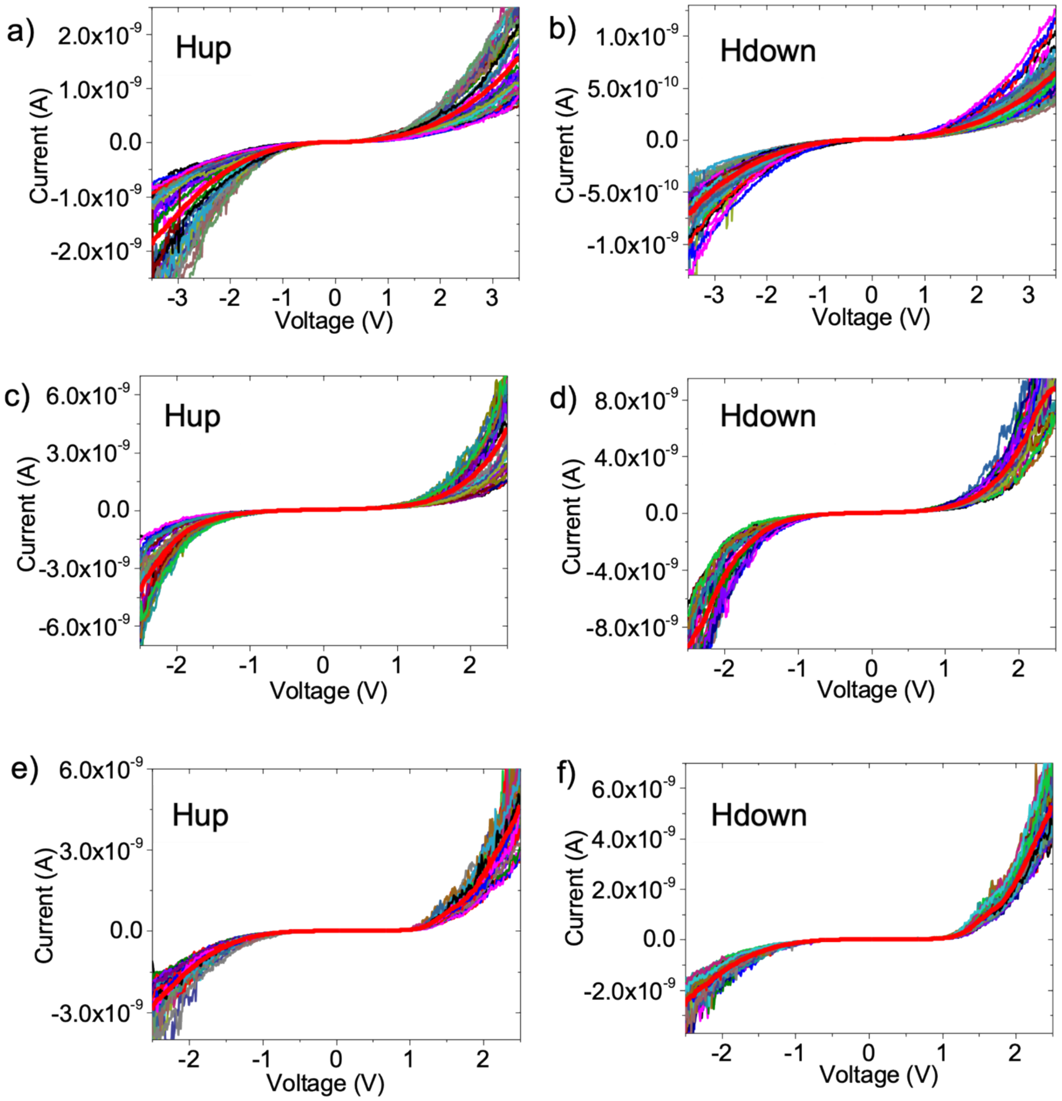

Figure S2. Current versus voltage (I-V) plots recorded for spin-coated of **SubPc 2** on Ni-Au substrates. (a-b) ***M*** enantiomer, (c-d) ***P*** enantiomer, (e-f) racemic mixture. In all cases, the Ni substrates were magnetized with the north pole pointing in the up or down orientations as indicated in each case. The width of the lines (in multiple colours) represents the standard deviation of the measurements.
